# The impact of classroom seating location and computer use on student academic performance

**DOI:** 10.1371/journal.pone.0236131

**Published:** 2020-08-05

**Authors:** Paris Will, Walter F. Bischof, Alan Kingstone

**Affiliations:** Department of Psychology, University of British Columbia, Vancouver, BC, Canada; Indiana University Bloomington, UNITED STATES

## Abstract

A student’s ability to learn effectively in a classroom setting is subject to many factors. While some factors are difficult to regulate, this study explores two factors that a student, or instructor, has full control over, namely 1) seating position, and 2) computer usage. Both factors have been studied considerably with regard to their effects on student performance, and the results indicate that sitting further from the instructor, or using a computer in the classroom, are related to a decline in grade performance. However, it is unclear if the choice of where to sit and whether or not to use a computer in class are mediated by the same cognitive process. If they are the same, then we would expect to see an interaction between the factors, such that, for example, computer usage would most negatively impact the grades of students who sit near the back of a class. This study aims to answer this question by looking at the individual and combined effects of seating position and computer usage on classroom performance. We sampled 1364 students, collecting nearly 3000 total responses across 5 different introductory psychology courses with 4 different instructors on 3 separate occasions. In agreement with previous research, we found that sitting further from the instructor negatively impacted students' grades (0.75 percentage points/row), and using a computer in class negatively impacted grades (by 3.88 percentage points). Our novel finding is that these deleterious effects combined in an additive manner, such that using a computer had the same harmful effect on grade performance regardless of whether the student sat at the front or back of the classroom.

## Introduction

There are a variety of factors that may influence student performance in classrooms, such as study habits, sleep, nutrition, and family and peer support. However, a student’s and/or instructor's ability to affect positive change is compromised by a number of factors, not the least of which being that these and many variables like them, operate beyond the classroom itself. There are, however, two factors that may have a significant impact on class performance that are relatively easy to implement within the classroom itself—seating position and the use of technology. As we review below, empirical investigations point to the general conclusion that students perform better when they sit closer to the front of the classroom; and students perform better when they are not using a laptop computer in class (or some comparable device, e.g., a tablet or phone).

### Seating position

The preference for seating location appears to be affected by multiple factors. As Tagliacollo et al. [1] found, sitting closer to the front of the classroom is correlated with fewer absences and better classroom performance, which they attribute to a higher motivation for learning. Similarly, Shernoff et al. [2] conclude that seating preferences in large courses are mediated by student engagement, as well as by learning orientation, classroom self-esteem, and intrinsic motivation to learn.

Indeed, studies dating back to the 1970’s have found that students sitting in the front of the classroom tend to perform better than those sitting in the back [3–8]. Zomorodian et al. [5], for example, analyzed seating position and final exam scores, and found that students who sat in the high-interaction zone (i.e., close to the front of the class) had better final exam scores.

What is not clear from this, and similar work [1–8], is why seating position and performance are related. It could be that 'better students' choose to sit in the front and 'poorer students' choose to sit in the back, rendering their relationship correlational rather than causal. For example, Blume et al. [8], who randomly assigned students to either a proximal or distant location to the teacher in a virtual classroom, found that most students learned better when sitting closer to the teacher, although students displaying ADHD symptoms did not get a learning benefit from closer proximity to the teacher. Nevertheless, it is worth noting that a survey study by Benedict and Hoag [9] did suggest that students who preferred to sit in the back but reported being 'forced' to move closer because their preferred seat was unavailable, tended to do better in class.

To try to get at this issue directly, Perkins and Wieman [10] assigned students randomly to a seat location in the first half of a physics course and switched the seating locations in the second half of the term, such that those in the front were seated in the back and vice versa. The seating location assigned for the first half of the term had an impact on students’ success in the course, in particular when the top and bottom ends of the grade distribution were considered: students who were initially sitting closer to the front had a higher proportion of grades in the top 20% compared to the students in the back, and they had a lower proportion of grades in the bottom 10% compared to the students in the back. However, the reversal of the seating order for the second half of the term did not reverse this trend. The implication is that seating position 'sets the tone' for a student's performance, such that being in the front is beneficial, and being in the back is detrimental, and once established, this cannot be undone in the classroom. However, in an attempt to replicate the results of Perkins and Wieman [10], Kalinowski and Taper [11] found no difference in course performance based on assigned seating location. Similarly, Meeks et al. [12] attempted to replicate the findings of Perkins and Wieman [10], but found that student performance was not significantly altered by assigned seating location over a ten-year longitudinal study.

In sum, there is good evidence that students who choose to sit nearer to the front of the classroom will do better than those who sit in the back, but the evidence is mixed, at best, that one can make students perform better or worse, by moving them from the back to the front, or vice versa. Collectively, these studies point to the importance of factors such as student motivation and interest as driving seat location and performance, rather than the reverse.

### Computer use

Many studies have reported a negative effect of laptop use (or the use of digital devices in general) on classroom performance [13–19]. For example, Aguilar-Roca et al., [15] divided the classroom into laptop-permitted zones, laptop-banned zones, and control zones where the use of a laptop was not regulated. There was no difference in attendance or percentage of students using a laptop between zoned and control sections, but there was a correlation between academic performance and note-taking preference (paper versus laptop), with paper note takers scoring significantly higher, and laptop users scoring significantly lower, than predicted by pre-class academic indicators. Glass and Kang [19] permitted or disallowed the use of digital devices (laptops or cellphones) in certain classes and found that there was no difference in clicker question scores between classes that permitted technology use and those that banned it. However, they found long-term effects, specifically that exam performance was worse for material that was taught during a technology permitted class. Although the above studies manipulated laptop and/or other technology opportunities within the classroom, it need not follow that those opportunities were always, or even often, taken up. For instance, in laptop allowed sections, students may have preferred to use pen and paper rather than a laptop. Similarly, in the laptop banned classrooms, students might be able to use a smartphone or some other form of digital technology. Note, however, that the lone exception to this appears to be the study by Glass and Kang [19] who applied the additional experimental control of having a proctor in the classroom to monitor and enforce the no technology rule.

In order to explore the mechanism underlying the negative effect of laptop usage, several investigators have focused on the type of laptop usage that students engaged with when using their devices, namely off-task (distractive) activities versus on-task (academic) activities. Kraushaar and Novak [20] found that a higher proportion of distractive activities in class leads to lower academic performance. Similarly, Bellur et al. [18] reported that students who engaged in off-task technology usage in the classroom had lower grade point averages. Ravizza et al. [21] monitored students’ internet usage during course lectures over a term and found that non-academic internet usage during class correlated negatively with the final exam score, but academic internet usage was not related to the final exam score. Finally, Wu, Mei and Ugrin [22] analyzed cyberloafing (leisurely internet usage) in and out of class and found that in class cyberloafing correlated negatively with student performance, whereas moderate out of class cyberloafing led to better performance.

Some authors have also investigated–with mixed results–how the use of laptops affects neighboring students in the classroom. Aguilar-Roca et al. [15] found that laptop use did not affect performance of surrounding students. In contrast, Fried [14] found that laptop usage was distracting to fellow students, and Sana et al. [17] found that students who were in direct view of a laptop also scored lower on a test, indicating that laptops may pose a distraction for fellow students. A recent study by Rana et al. [23] may explain why these mixed results occur. Specifically, it appears that in-class norms may play a critical role. While neighbor distraction alone may not encourage students to engage in cyberloafing, it may increase the perception of in-class norms and thus cause more students to engage in this behaviour. The use of laptops may also have other deleterious effects, such as a decrease in student engagement in class [24], and the contribution to inefficient study habits [18].

### Combined effects

What is far from clear is whether the two factors of seating preference and computer usage are mediated by the same or different cognitive mechanisms, as no study to date has systematically set out to test this issue. One can well imagine that students who sit in the back of the class and *also* use a computer will be especially compromised. There is a small hint, however, that this is not the case. A recent, excellent study by Wammes et al. [25] found that media multitasking (e.g., laptop use), but not mind wandering, had a negative effect on learning performance. And in a largely exploratory analysis, they compared performance as a function of seating location. While those in the back of the classroom used computers significantly more than those in the front of the classroom, this did not have a significant effect on learning performance (though there was a numerical difference with those in the back performing worse). Moreover, for a small number of students who sat *both* in the back and the front of the class over the duration of the course, there was no effect of seating location on computer use, mind wandering, or test performance. These findings suggest that the effects of seating location and computer use are driven by different cognitive processes. Though, we should note that the authors were adamant that their results on this issue were only exploratory in nature and were included merely to guide future work. The aim of the present study is to pick up this baton.

The present study explicitly examines the combined effects of seating position and computer use. Applying Sternberg's additive factors method, we predicted that if both factors are mediated by the same cognitive process, then the effects of seating position and computer use should interact, i.e., one would expect students who sit near the front of the class to be affected the least by computer use, and those who sit at the back to be impacted the most. On the other hand, if the mechanisms underlying the two factors are independent, then one expects the effect of the two factors to combine in an additive manner. There are, however, some important limits to this additive factors logic, which we will explore further when discussing our results. For now, we note simply that it *is* possible that if the factors of seating position and computer use combine in an additive manner, their individual influence on academic performance may be independent, but the cognitive mechanism underlying where one chooses to sit and whether one does or does not use a laptop, may be the same.

In the previous studies outlined above, with the exception of two studies [8, 17], it is unclear whether the instructors were involved in conducting the experiment. The present study utilizes an instructor naivety method, where the classroom instructors are both unaware of the purpose of the study and they are not involved in data collection. To control further for the effects of instructor and to ensure that the seating position data are reliable, we measured seating location and computer use in five large first-year university classes (some with the same instructor and others with different instructors) on three separate occasions over the course of a 13-week university term, collecting nearly 3000 student observations. To anticipate our findings, the data are unequivocal—students perform worse academically when they are further from the instructor and they do worse when they are using a computer in class. These two findings converge with past investigations that have examined either sitting position or computer use in class. Our novel finding is that these two factors combine in an additive manner, e.g., the detrimental effect of a computer is the same when it is used in the front of the class where students in general perform better, and in the back of the class where students generally perform worse.

## Methods

### Participants

The present study sampled across five separate introductory psychology courses over one semester at the University of British Columbia (UBC). Data were collected from each course on three separate occasions throughout the term, with a separation of 4 weeks between each data collection. Course rosters indicated a total of 1364 registered students across all five courses, but due to variance in daily attendance, we collected data from 1151, 925, and 855 students on the first, second, and third instances of data sampling. All students consented to participating in the present study and were assured that data were treated confidentially and did not influence course grades.

### Course structure and lecture hall

All five introductory psychology courses followed the same weekly schedule, such that classes were fifty-minutes in length and held every Monday, Wednesday, and Friday; four of the five classes were taught by different instructors (i.e., one instructor taught two sections). In none of the five courses were computers (e.g., laptops, tablets, phones) mandatory, but students were allowed to use computers to take notes.

All course sections were taught in the same modern lecture hall with a seating capacity of 450 in UBC’s Centre for Interactive Research on Sustainability (CIRS). Lecture seating is arranged into 18 rows of varying length and divided into front (rows 1–5), middle (rows 6–15), and back (rows 16–18) regions, with regions separated by aisles and further split in half by an aisle running down the middle of the hall. In addition to fixed seating, the lecture hall contains two five-person tables located in the fourth row from the lectern.

### Data collection

Ethics approval for this study was obtained from the University of British Columbia’s Behavioural Research Ethics Board (BREB). In the final 10 minutes of class, researchers introduced themselves and provided a brief description of the current study’s intent to investigate migration patterns in student seating in the CIRS building; however, special effort was made to avoid drawing attention to the impact of seat location and computer use on student performance. The researchers then distributed paper handouts, numbered by seating row, and students were required to indicate their seat number, initials, last five digits of their student number, and Yes or No as to whether or not they used a laptop computer or some other electronic device (e.g., a phone or tablet) for note taking. Data collection was conducted on three separate occasions during the semester, at roughly one-month intervals, beginning one-month after the start of the semester. Data was collected on Fridays with the exception of one collection for a class on a Wednesday. The classes were conducted at different times, starting on the hour, between 9am-1pm. Further, for any given round of data collection, all course sections were sampled within seven days of one another. Academic performance was calculated at the end of the semester based on the student’s final grade in the course. In all sections, final grade was composed of two mid-term exams and a cumulative final exam, each contributing equally to the final grade.

### Data analysis

Over the three rounds of data collection, 2914 responses from the 1364 students were collected, and 2662 (95%) were usable for our analyses. Multiple linear regression analyses were used to assess the effect of row, gender, and computer use on students’ grade. All classical statistical analyses were performed using Stata 15.1 [26]. Bayesian analyses were performed using Stata and JASP [27], are interpreted according to Lee and Wagenmakers [28], and are reported in brackets after the classical statistical results.

## Results

**Fig 1:**
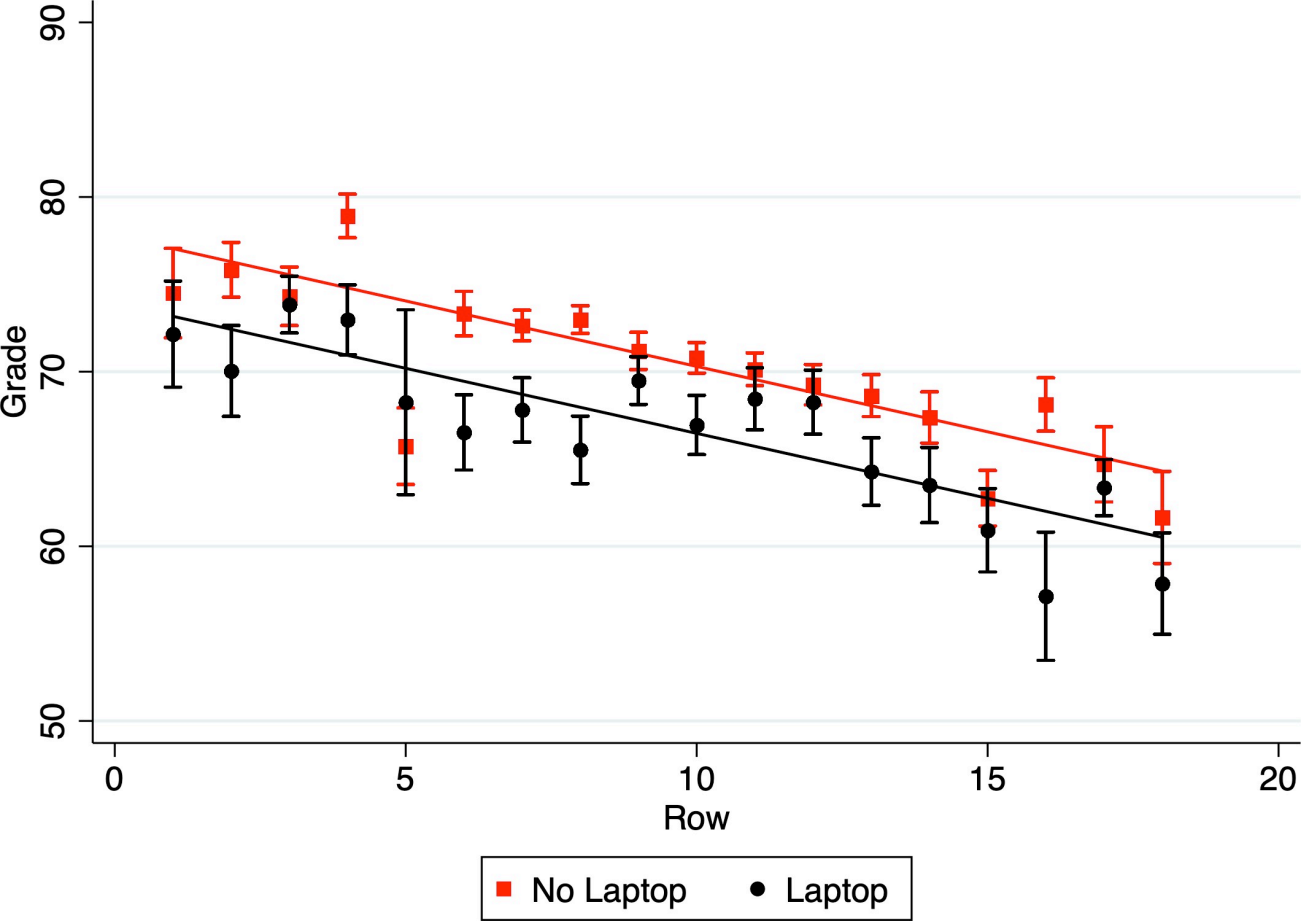
Relationship between grade, row and computer. Performance (Grade) is plotted against row in the lecture room, separately for students who used a computer and those who did not. For each row, the mean grade and standard errors are shown.

A multiple linear regression was calculated to predict performance in the course (Grade, in percentage points) based on seating position (Row, 1–18) and computer use (Computer, Yes/No). A significant regression was found (*F*(3,2658) = 63.64, *p* < .001), with an *R*^*2*^ of .067. [In addition, the data were examined by estimating a Bayes factor using Bayesian Information Criteria. The data were fit with a full regression model grade = f(row, computer, row × computer) and compared to a null model. The estimated Bayes factor *BF*_10_ = 4.85·10^31^ suggests that the data are 4.85·10^31^ more likely to be observed under the full regression model H_1_ than under the null model H_0_, thus providing extreme evidence in favor of the regression model H_1_.] The effect of Row was significant (*t*(2660) = -9.85, p < .001, *η*^*2*^ = 0.034), with the average grade decreasing by 0.75 percentage points with each row that students sat farther away from the lecturer. [The estimated Bayes factor *BF*_10_ = 1.76·10^27^ suggests that the data are 1.76·10^27^ times more likely to be observed under H_1_ than under H_0_, thus providing extreme evidence for H_1_.] The effect of Computer was also significant, (*t*(2660) = -2.79, p = 0.005, *η*^*2*^ = 0.003), with the average grade decreasing by 3.88 percentage points if a laptop was used. [The estimated Bayes factor *BF*_10_ = 7.44·10^7^ suggests that the data are 7.44·10^7^ times more likely to be observed under H_1_ than under H_0_, thus providing extreme evidence for H_1_.] There was no interaction between Row and Computer (*t*(2660) = 0.04, p = .971) (see Fig 1). The data were fit with an additive model and compared to an alternative, interactive model that included a Row × Computer interaction term. The estimated Bayes factor between interactive and additive model, *BF*_IA_ = 0.001336, suggests that the data were 748 times more likely to occur under the additive than the interactive model, thus providing extreme evidence for the additive model.] We also found that the probability of using a computer was not affected by seating position (Wilcoxon rank-sum *z* = -1.65, *p* = .099). [The estimated Bayes Factor *BF*_10_ = 0.096 suggests that the data are 10.4 times more likely to be observed under H_0_ than under H_1_, thus providing strong evidence for H_0_.]

Other potentially influencing factors were found to have no effect of course performance: There was no effect on performance if either the neighbor on the left or on the right used a computer (*t*(1801.3) = 1.42, *p* = .153, using Satterthwaite’s degrees of freedom). [The estimated Bayes Factor *BF*_10_ = 0.141 suggests that the data is 7.1 times more likely to be observed under H_0_ than under H_1_, thus providing moderate evidence for H_0_.] Furthermore, a multiple linear regression to predict Grade based on Row, Computer and Instructor yielded no significant three-way interaction of Row × Computer × Instructor, indicating that the effects reported here were not affected by the instructors (and their teaching style). [In the Bayesian analysis, three regression models were compared, namely the additive model A grade = f(row, computer), model B, which included an instructor term, grade = f(row, computer, instructor), and model C, which included an instructor term plus interactions, grade = f(row, computer, instructor, row × instructor, computer × instructor). The Bayes factor of the comparison of models A and B, *BF*_BA_ = 3·10^−^7, indicated that the data were extremely in favour of model A, and the Bayes factor of the comparison of models A and C, *BF*_CA_ = 5·10^−29^ also indicated that the data were extremely in favour of model A. Together, the analyses provided extreme evidence against an instructor effect.] Finally, students’ seat location remained largely fixed throughout the term (i.e., there was low seating migration between lectures), and the interaction between seating migration and grade was found to be nonsignificant (*F*(1,2660) = 0.32, *p* = .572). [The estimated Bayes factor of the interaction between seating migration and grade, *BF*_10_ = 0.051, suggests that the data are 19.6 times more likely to be observed under H_0_ than under H_1_, thus providing strong evidence for H_0_.]

Finally, the average grade of men (*M* = 68.0) was significantly worse than that of women (*M* = 71.3) (*t*(1839.2) = 6.21, *p* < .001, Cohen’s *d* = 0.26, using Satterthwaite’s degrees of freedom). This difference can—at least partially—be understood from the fact that men were on average sitting further back in the classroom than women (Wilcoxon rank-sum *z* = -6.58, *p* < .001). [The estimated Bayes factor, *BF*_10_ = 85.5, indicates that the data are 85.5 times more likely to be observed under H_1_ than under H_0_, thus providing strong evidence for H_1_.] Men were marginally more likely than women to be using a computer (*χ*^2^(1) = 3.55,*p* = .060). [The estimated Bayes Factor, *BF*_10_ = 0.272, suggests that the data are 3.68 times more likely to be observed under H_0_ than under H_1_, thus providing moderate evidence for H_0_.]

## Discussion

The goal of every instructor is to maximize the learning experience for the students in their class. However, many of the factors that are correlated with learning performance, such as intrinsic motivation, interest in the material, and good sleep and nutrition, are outside the direct control of instructors. The literature suggests that there are two factors shown to influence the learning performance of students, and these are where a student sits and whether or not they use a computer in class.

To date there have not been any studies that directly set out to test the relationship between seating position and computer use, although there was a recent exploratory report [25] tentatively suggesting that computer use and sitting position do not interact. However, as Wammes et al. [25] hasten to note, their evidence on this issue was purely exploratory, being drawn from an analysis of less than 70 students.

The present study sought to examine this issue directly, collecting data in five different lecture sessions, with four different instructors, on three different occasions, each separated by four weeks: 1151 students on our first test session, 925 students on the second session, and 855 students on the third session. Thus, we had excellent power to detect the effect of seating position and computer use.

As expected, we found a significant effect of seating position, with every row that students sat away from the lecturer being associated with an average grade decline of a 0.75 percentage point. We also found an effect of computer use, with participants’ average grade dropping by 3.88 percentage points when they used a computer in class. Note also that we found that computer use did not have a negative effect on the performance of neighboring students. This is consistent with the results of Aguilar-Roca [15], but stands in contrast with the results found by Fried [14] and Sana et al. [17]. The reason for these mixed findings may be based on different measures used to indicate computer distraction. While our study looked at left/right neighbouring student’s computer use as a source of potential distraction, the other studies compared performance between zoned sections [15], self-report distraction [14], or distraction from frontal student’s [17]. Furthermore, based on the work of Rana et al. [23] we can infer that in-class norms in our study may have discouraged cyberloafing.

Most importantly, and as clearly indicated by Fig 1, the effect of seating position and computer use combine in an additive manner. That is, whether one is a student sitting in the front of the class, or a student sitting in the back of the class, the impact of using a computer is to drop one's grade by nearly 4 percentage points. At our university, that is equivalent to a change in a letter grade, e.g., an A- to a B+.

Note that the independence of these two factors—seating position and computer use—was suggested by classical null hypothesis testing, and critically, complemented by Bayesian analysis, with the Bayes factor indicating that our data were 748 times more likely to occur under a model of additivity than interactivity, thus providing extreme evidence for independence between the two factors. According to Sternberg's additive factors logic, independence of factors suggests that they are mediated by separate cognitive processes. What might those different processes be?

The literature on classroom seating choice indicates that students who choose to sit in the front of the classroom score higher on a measure of achievement motivation [29]. This work also reveals that student achievement motivation scores increase throughout the course regardless of where they sit. This suggests that pre-dispositional motivation may play an important role in the effect of seating location on academic performance. Further, individual differences such as personality have been explored, with Totusek and Staton-Spicer [30] finding personality differences between students who choose to sit in action seats (e.g., near to the instructor) versus non-action seats. Students closer to the instructor in the action seats have significantly higher levels of a personality dimension classified as imaginative [30], which includes one’s creativity, absorption in ideas, and unconventional approach to life. If these individual differences underlie a student’s choice in seating position, then what drives a student’s decision to use a laptop in class? To our knowledge no study has directly examined the factor(s) underlying a student’s choice of using a computer in class. However, related research in regards to one’s ability to embrace and use technology suggests that individual differences are likely to be involved. For instance, Parasuraman [31] found that individuals scoring higher on technology related innovative personality traits are more likely use new technologies, which is enhanced further by a technology's perceived usability [32]. In addition to these personality traits, non-personality related differences, such as access to technology, may also drive this decision.

It should be noted that recent work suggests that within Sternberg's additive factors framework, independence of two factor need not demand the conclusion that the processes are mediated by distinct cognitive processes [33]. Thus, the academic outcomes of seating choice and computer use may be independent, but it is possible that the decision about where to sit and whether to use a computer are being driven by the same cognitive process, such as a desire to do well in class. Thus, having demonstrated their independent effect on academic performance, our work presents the opportunity for a future investigation to directly measure the cognitive processes involved in a student's decision of seating location and computer (or other forms of technology) use.

We began our study by raising the possibility that there are two ways that instructors may be able to positively affect the learning experiences of students. The evidence suggests that the effect of seating position, though significant, may be outside of an instructor's control, as it appears to be driven by internal variables that students bring to class, e.g., their motivation and personality. However, if the cognitive process for the decision to use a computer is independent of the decision of where one sits, and there is a connection between computer use and grade performance, then it is possible that instructors may be able to have a positive effect on students' learning experience by discouraging them from using computers during class. Although our study is well-powered—sampling a wide range of instructors and students—it remains be confirmed that our results extend to other disciplines and institutions. If they do, then it raises the real possibility that the decision to use a computer in class may make the difference between whether a student gets an A or a B, or a pass or a fail.

## Supporting information

S1 Raw Data (TXT)
